# A new method to improve RF safety of implantable medical devices using inductive coupling at 3.0 T MRI

**DOI:** 10.1007/s10334-023-01109-8

**Published:** 2023-08-11

**Authors:** Bu S. Park, Joshua W. Guag, Hongbae Jeong, Sunder S. Rajan, Brent McCright

**Affiliations:** 1 Division of Cellular and Gene Therapies (DCGT), OTAT, CBER, Food and Drug Administration (FDA), Silver Spring, MD USA; 2 Division of Biomedical Physics (DBP), OSEL, CDRH, FDA, Silver Spring, MD USA

**Keywords:** SAR, FDTD, Computational modeling, Implantable medical device, Secondary resonator, Inductive coupling

## Abstract

**Objective:**

To enhance RF safety when implantable medical devices are located within the body coil but outside the imaging region by using a secondary resonator (SR) to reduce electric fields, the corresponding specific absorption rate (SAR), and temperature change during MRI.

**Materials and methods:**

This study was conducted using numerical simulations with an American Society for Testing and Materials (ASTM) phantom and adult human models of Ella and Duke from Virtual Family Models, along with corresponding experimental results of temperature change obtained using the ASTM phantom. The circular SR was designed with an inner diameter of 150 mm and a width of 6 mm. Experimental measurements were carried out using a 3 T Medical Implant Test System (MITS) body coil, electromagnetic (EM) field mapping probes, and an ASTM phantom.

**Results:**

The magnitudes of **B**_1_^+^ (|**B**_1_^+^|) and **SAR**_**1g**_ were reduced by 15.2% and 5.85% within the volume of interest (*VoI*) of an ASTM phantom, when a SR that generates opposing electromagnetic fields was utilized. Likewise, the Δ|**B**_**1**_^**+**^| and Δ**SAR**_**1g**_ were reduced by up to 56.7% and 57.5% within the *VoI* of an Ella model containing a copper rod when an opposing SR was used.

**Conclusion:**

A novel method employing the designed SR, which generates opposing magnetic fields to partially shield a sample, has been proposed to mitigate the risk of induced-RF heating at the *VoI* through numerical simulations and corresponding experiments under various conditions at 3.0 T.

## Introduction

Magnetic resonance imaging (MRI) is widely used for clinical applications because it results in images with excellent soft tissue contrast and does not use ionizing radiation. Although MRI does not emit ionizing radiation associated with other non-invasive imaging methods such as X-ray computed tomography (CT), the electromagnetic (EM) fields from MRI can pose thermal risks when high level of EM-fields is absorbed by the tissue. This is particularly concerning for those patients with implanted medical devices, which can accumulate EM-fields in regions of the body surrounding the implants.

Studies have been conducted to evaluate the risks associated with MRI scanning on patients with implantable medical devices such as implantable deep brain stimulators (DBS) [1, 2], pacemakers [3], cardioverter-defibrillators (ICDs), stents [4, 5], fracture fixation screws [5], and breast tissue expander devices [6]. For example, Ting Song et al*.*, demonstrated radiofrequency (RF) induced heating values using the American Society for Testing and Materials (ASTM)-based RF heating data from 86 premarket U.S. Food and Drug Administration (FDA) 510(k) cleared submissions at 1.5 T and 3.0 T [5]. In the study, the local background (LB)/whole-body (WB) SAR ratio varied from 2.3 to 11.3 for a given WB SAR, and the maximum temperature rise for stents was measured at stent lengths of approximately 100 mm at 3.0 T, and beyond 150 mm at 1.5 T [5].

A significant RF-induced temperature rise or high **SAR** values can cause safety concerns in human studies and should be addressed based on the several guidelines [7–9]. Specifically, the International Electrotechnical Commission (IEC) [7] and the FDA have issued guidelines to limit the **SAR** values of local **SAR**, 1 g-averaged **SAR** (**SAR**_**1g**_), and 10 g-averaged **SAR** (**SAR**_**10g**_), as well as the temperature rise during human MRI procedures [8]. According to the IEC guidance [7], the local **SAR** limits are 10 W/kg for head and trunk, and 20 W/kg for the extremities, averaged over a period of 6 min in normal operating mode. Furthermore, ISO/TS 10974 has been developed to provide test methods to evaluate the safety of active implantable medical devices (AIMDs) for MRI [9].

Several methods have been proposed to improve RF safety in MRI, both with and without the use of AIMDs. One such approach utilizes multiple transmit sources, referred to as a parallel transmit-array (TA) [10, 11]. In this method, the amplitude and phase of each channel are optimized individually, either to decrease **SAR** values or to improve the uniformity of transverse magnetization (**M**_**t**_).

Another method involves the use of high dielectric constant (HDC) materials, also known as high-permittivity materials (HPM). These materials modify EM-fields within the volume of interest (*VoI*) to improve transmit efficiency, signal-to-noise ratio (SNR), or reduce SAR during MRI [12–14]. The advantage of this method is that it does not require modifications to existing MRI systems. However, challenges for adopting this approach include the high cost and complexity of producing HDC materials, as well as the limited space around the RF resonator.

Another potential approach to reduce SAR from implantable medical devices involves modifying the EM fields using inductive coupling with loop resonators [15]. In a study by Merkel et al. [15], the authors demonstrated that the actively controlled, off-resonant loop elements could enhance local RF magnetic fields (**B**_**1**_) in the *VoI*. This was achieved when the loop elements were tuned to enhance magnetic fields in comparison to the fields produced by the volume transmit coil. The results, which included enhanced **B**_**1**_ and SNR in the *VoI*, were consistent with those found in many previous studies on inductive coupling [16–18]. While the study primarily focused on improving magnetic field uniformity and SNR, the loop elements can also be tuned to produce opposing magnetic fields and reduce RF exposures at a specific target location. This could offer simple and cost-effective ways to mitigate the risks associated with RF exposure during an MRI.

Building on previous research [15, 18], we propose a new method to enhance RF safety when implantable medical devices are located within the body coil but outside the imaging region. We describe the design and application of a SR that can be used to reduce the electric fields and heating in the region surrounding the implants. This would apply, for instance, when a patient with implants in the shoulder or knee undergoes heart imaging using MRI. Numerical simulations of SR effects were conducted using both the ASTM phantom and human models, followed by corresponding experimental verifications with the ASTM phantom. The EM effects of the SR on a copper rod representing a medical stent were also evaluated [4].

## Theory

**Fig. 1:**
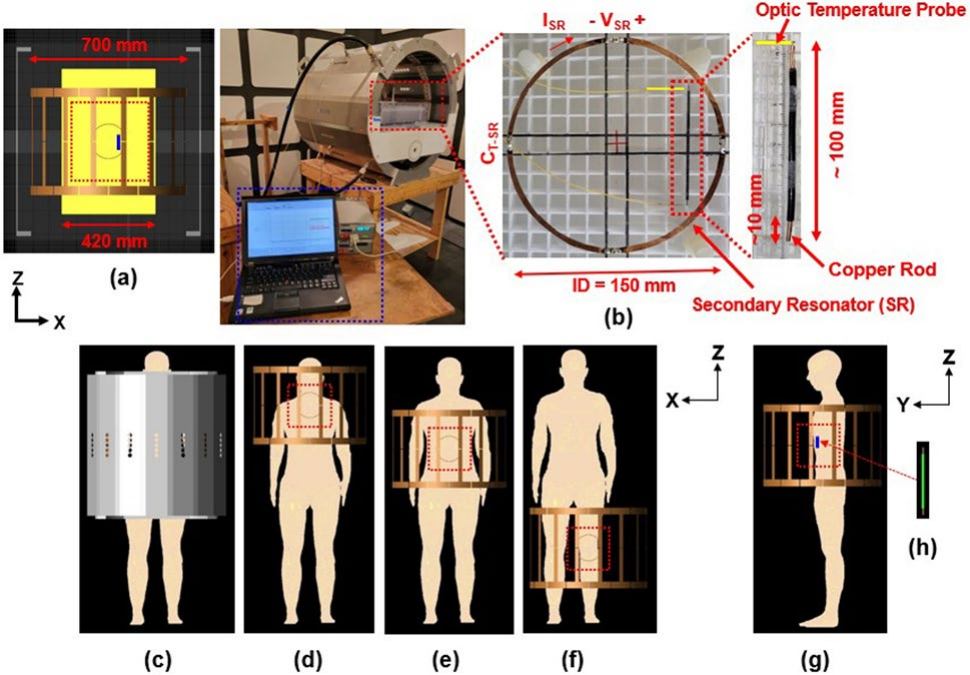
Geometrical models used for this study. **a** Model of the 16-leg bandpass (BP) birdcage body coil (brown), ASTM phantom (yellow), circular secondary resonator (grey), and copper rod (L = 100 mm, blue line) at 3.0 T (128 MHz); **b** Experimental setup with a designed SR, temperature measurement system including an optic temperature probe (yellow line), and a copper rod; **c** Ella model with RF shield, 16-leg BP birdcage coil and SR with different landmark positions of **d** neck, **e** sternum, and **f** knee; The side view of Ella model with a copper rod ((blue line in **g**) and **h**). (Red dotted rectangular boxes represent the volume of interest (*VoI*). Another human model of Duke used in this study is not shown in this figure)

Based on Faraday’s induction law, the electromotive force (EMF) around a SR is equal to the negative time derivative of the external magnetic flux passing through it as follows [19],

1 $$\mathrm{EMF}={\mathbf{V}}_{\mathrm{SR}}\left(\mathrm{t}\right)= -\frac{\mathrm{d\varnothing }}{\mathrm{dt}}= -\frac{\mathrm{d}}{\mathrm{dt}}\iint {\mathbf{B}}_{1\_\mathbf{V}\mathbf{T}}\left(\mathrm{t}\right)\cdot \mathrm{d}\mathbf{S}$$

2 $${\mathbf{B}}_{1\_\mathbf{S}\mathbf{R}}\propto {\mathbf{I}}_{\mathrm{SR}}\propto \frac{1}{{\mathbf{Z}}_{\mathrm{SR}}}$$

3 $$\nabla \times {{\varvec{E}}}_{{\varvec{i}}}= -\frac{\partial {{\varvec{B}}}_{1}}{\partial t}$$

where ${\mathbf{V}}_{\mathrm{SR}}$ is the potential difference in the SR (V), ϕ is the magnetic flux (Wb), ${\mathbf{B}}_{1\_\mathbf{V}\mathbf{T}}$ is the RF magnetic field (T or Wb/m^2^) made by the volume transmit coil, i.e., body coil in our study, ${\mathbf{B}}_{1\_\mathbf{S}\mathbf{R}}$ is the induced RF magnetic field (T or Wb/m^2^) made by the SR, ${\mathbf{I}}_{\mathrm{SR}}$ is the induced current (A) in the SR, ${\mathbf{Z}}_{\mathrm{SR}}$ is the total impedance (Ω) of the SR, and ${{\varvec{E}}}_{{\varvec{i}}}$ is the magnetically-induced electric field (Fig. 1b).

Therefore, it is possible to configure **Z**_**SR**_ as an equivalent inductor or capacitor, exhibiting a 180-degree phase difference, by using the tuning capacitor in the SR (i.e., C_T-SR_, as depicted in Fig. 1b). This results in two different induced currents flowing in opposite directions.

The ${\mathbf{I}}_{\mathrm{SR}}$ and related ${\mathbf{B}}_{1\_\mathbf{S}\mathbf{R}}$ can be calculated under the following assumptions: (1) both the body coil and the SR have negligible resistance, and (2) there is no strong coupling between the body coil and the SR.

Case 1) Enhancing ${\mathbf{B}}_{1\_\mathbf{S}\mathbf{R}}$: f_SR_ > f_o_ = 128 MHz

4 $${\mathrm{Z}}_{\mathrm{SR}}\approx \frac{1}{j{\omega }_{SR}{C}_{eq}},{\mathrm{ I}}_{\mathrm{SR}}\approx {C}_{eq}\frac{d{\mathrm{V}}_{\mathrm{SR}}}{dt}$$

where ${\omega }_{SR}$ is the radial frequency of the SR (${\omega }_{SR}=2\times \pi \times {f}_{\mathrm{SR}})$, f_o_ is the resonance frequency of the body coil, and C_eq_ is the equivalent capacitance value (F) of the SR.

In this case, the ${\mathbf{I}}_{\mathrm{SR}}$ leads ${\mathbf{V}}_{\mathrm{SR}}$ and ${\mathbf{B}}_{1\_\mathbf{V}\mathbf{T}}$ by 90° making ${\mathbf{B}}_{1\_\mathbf{S}\mathbf{R}}$ in-phase with ${\mathbf{B}}_{1\_\mathbf{V}\mathbf{T}}$ resulting in enhancing fields.

Case 2) Opposing ${\mathbf{B}}_{1\_\mathbf{S}\mathbf{R}}$: f_SR_ < f_o_ = 128 MHz

5 $${\mathrm{Z}}_{\mathrm{SR}}\approx j{\omega }_{SR}{L}_{eq},{\mathrm{V}}_{\mathrm{SR}}\approx {L}_{eq}\frac{d{\mathrm{I}}_{\mathrm{SR}}}{dt}$$

where L_eq_ is the equivalent inductance value (H) of the SR.

In this case, ${\mathbf{V}}_{\mathrm{SR}}$ leads ${\mathbf{I}}_{\mathrm{SR}}$ by 90°, which gives ${\mathbf{B}}_{1\_\mathbf{V}\mathbf{T}}$ a negative sign. Consequently, ${\mathbf{B}}_{1\_\mathbf{S}\mathbf{R}}$ has an opposite direction compared to ${\mathbf{B}}_{1\_\mathbf{V}\mathbf{T}}$, resulting in opposing fields. The principle of Case 2) is primarily utilized in this study. Detailed calculation procedures for these equations can be found in previous research [15, 18].

## Materials and methods

This study utilized numerical simulations based on the Finite Difference Time Domain (FDTD) method [20] and computational models of a body transmit coil (which generates circularly polarized **B**_1_ fields), a SR, an ASTM phantom [21], and two adult human models: Ella and Duke [22]. Corresponding experiments using the ASTM phantom were conducted for experimental verification. The simulations were employed to evaluate the RF magnetic field’s X-component (**B**_**X**_) and Y-component (**B**_**Y**_), the rotating RF magnetic field in the transmit mode (**B**_1_^+^ = (**B**_X_ + j**B**_**Y**_)/2), and the SAR distributions at 128 MHz, which corresponds to the 3.0 T MRI proton frequencies (Fig. 1a and c).

### Numerical simulations

#### Computational model of body coil, SR, and ASTM phantom

A quadrature 16-leg band-pass (BP) body coil model with an RF shield was utilized in the numerical simulations to assess the effects of the SR at 128 MHz. The BP body coil model features an inner diameter (ID) of 700 mm, inner length (L) of 400 mm, outer length of 480 mm, a copper strip width (W) of 40 mm for the end ring and 25 mm for each rod, as well as an RF shield (with ID = 830 mm, outer diameter (OD) = 837 mm, and L = 855 mm). These specifications were chosen in accordance with the experimental conditions.

The tuning capacitors (C_T_Body_) of the body coil were positioned in the end-rings, with values of 13.67 pF for the end-ring and 66.73 pF assigned to each rod (Fig. 1a).

The circular SR was designed with parameters of ID = 150 mm, and W = 6 mm. The center of the SR in the XZ-plane (Coronal view) aligned with the center of the body coil and ASTM phantom (Fig. 1a). The tuning capacitor used in the SR (C_T_SR_) was set at 6.5 pF for enhancing fields and 8.4 pF for opposing fields (Fig. 1b). These values were determined by checking the resonance frequency of the SR and the |**B**_**1**_^**+**^| distributions within the ASTM phantom acquired with the body coil, ASTM phantom, and the SR. The shortest distance between the ASTM phantom and the SR along the Y-axis was 24 mm.

The ASTM phantom was designed based on the ASTM standard test method [21] and has parameters of L = 650 mm, W = 420 mm, and height (H) = 90 mm. The center of the ASTM phantom aligned with the center of the body coil (Fig. 1a). The phantom’s electrical properties were set to a conductivity (σ) of 0.47 S/m and a relative permittivity (ε_r_) of 80 [6, 21].

#### Computational model of Ella, Duke, body coil, SR, and copper rod

The Virtual Family models of Ella and Duke [22] with the SR and copper rod were used in this study (Fig. 1). The Ella and Duke models have 36 different anatomical structures with electrical properties assigned as in the previous study [23–25]. Numerical simulations using the Ella model were performed at three different landmark positions: the Neck, Sternum, and Knee [26]. In contrast, for the Duke model, the simulations were performed only at the Sternum landmark position to evaluate the effects of the SR at different locations (Fig. 1d–f).

The circular SR having the same geometrical structures of ID = 150 mm, and W = 6 mm was used. However, the C_T_SR_ was changed to 12.0 pF for opposing fields and 6.5 pF for enhancing fields for all numerical simulations using human models including three different landmark positions of the Ella model in this study. The tuning frequency shift of the SR due to the landmark positions and different human models was negligible.

The shortest distance between Ella model and the SR was 15 mm for all three landmark positions.

A copper rod with a diameter = 3.8 mm, and L = 100 mm was used to evaluate the effect of the SR on the RF safety of medical implants [4]. The copper rod was used as model for medical devices such as stents.

#### FDTD numerical simulations

Multiple grid resolutions of 0.5 × 0.5 × 0.5 mm^3^ (copper rod region) and 3 × 3 × 3 mm^3^ (others) that had boundary conditions of 12 perfectly matching layers were used for the numerical simulations. The calculation time and accuracy of the numerical simulation was dependent on the resolution of the grid being analyzed.

The simulation results were normalized to the dissipated power to make |**B**_**1**_^**+**^| equal to 2.0 µT at the center of the body coil corresponding to a 90^ο^ flip angle of a 3 ms rectangular RF pulse [27] without the SR. Therefore, the dissipated power for each case would be different as follows: 48.84 W for the ASTM phantom study, 445 W (Sternum landmark without the copper rod), 611 W (sternum landmark with the copper rod), 83.4 W (neck landmark), 65.2 W (knee landmark) for the Ella study, and 1114 W for the Duke study.

The *VoI* was set as 180 × 180 × 180 mm^3^ (without the copper rod) or 132 × 132 × 135 mm^3^ (with the copper rod) to evaluate the effect of the SR. The center of *VoI* is the same as that of the SR and the body coil.

The |**B**_**1**_^**+**^|, |**B**_**Y**_| and **SAR**_1g_ over the *VoI* were computed. The **SAR**_1g_ refers to the average value of SAR in a 1-g region of tissue surrounding the voxel. The deviation of |**B**_**1**_^**+**^| (Δ|**B**_**1**_^**+**^|) and **SAR**_1g_ (Δ**SAR**_1g_) with and without the SR were calculated pixel wise as:

6 $$\Delta |{\mathbf{B}}_{1}^{+}|=\frac{{|{\mathbf{B}}_{1}^{+}|}_{\mathrm{With}}-{|{\mathbf{B}}_{1}^{+}|}_{\mathrm{Without}}}{\mathbf{M}\mathbf{e}\mathbf{a}\mathbf{n}{|{\mathbf{B}}_{1}^{+}|}_{\mathrm{Without}}}\times 100\boldsymbol{ }\;\left[\boldsymbol{\%}\right]$$

7 $${{\varvec{\Delta}}\mathbf{S}\mathbf{A}\mathbf{R}}_{1\mathrm{g}}=\frac{{\mathbf{S}\mathbf{A}\mathbf{R}}_{1\mathrm{g}\_\mathrm{With}}-{\mathbf{S}\mathbf{A}\mathbf{R}}_{1\mathrm{g}\_\mathrm{Without}}}{\mathrm{Mean}{\mathbf{S}\mathbf{A}\mathbf{R}}_{1\mathrm{g}\_\mathrm{Without}}}\times 100\;\left[\mathbf{\%}\right]$$

where Mean |**B**_**1**_^**+**^|_Without_ and ${\mathbf{S}\mathbf{A}\mathbf{R}}_{1\mathrm{g}\_\mathrm{Without}}$ are the mean value of |**B**_**1**_^**+**^| and **SAR**_1g_ over the *VoI* without the SR [6].

Max-, Min-, and Mean-${{\varvec{\Delta}}\mathbf{S}\mathbf{A}\mathbf{R}}_{1\mathrm{g}}$ (Tables 1, 2, 3 and 4) represent the maximum (Max ${{\varvec{\Delta}}\mathbf{S}\mathbf{A}\mathbf{R}}_{1\mathrm{g}}$), minimum (Min ${{\varvec{\Delta}}\mathbf{S}\mathbf{A}\mathbf{R}}_{1\mathrm{g}}$), and average (Mean ${{\varvec{\Delta}}\mathbf{S}\mathbf{A}\mathbf{R}}_{1\mathrm{g}}$) increase in ${\mathbf{S}\mathbf{A}\mathbf{R}}_{1\mathrm{g}}$ with the SR compared to that without the SR within the *VoI*. Negative Min ${{\varvec{\Delta}}\mathbf{S}\mathbf{A}\mathbf{R}}_{1\mathrm{g}}$ values in the Tables indicate the maximum decrease in ${\mathbf{S}\mathbf{A}\mathbf{R}}_{1\mathrm{g}}$ with the SR.

**Table 1:** “Without SR” vs. “with SR (enhancing)” vs. “with SR (opposing)” (ASTM phantom at 128 MHz, 700 mm BP body coil)

(A) |B_1_^+^| and Δ|B_1_^+^|							
	Max |B_1_^+^| [μT]	Mean |B_1_^+^| [μT]	Max Δ|B_1_^+^| [%]		Min Δ|B_1_^+^| [%]	Mean Δ|B_1_^+^| [%]	
Without SR	2.02	1.48	Reference	Reference			Reference
With SR (C_T-SR_ = 8.4 pF, opposing)	1.80	1.33	+ 47.8	− 94.9			− 17.6
With SR (C_T-SR_ = 6.5 pF, enhancing)	2.44	1.57	+ 75.3	− 29.4			+ 9.53

(B) SAR_1g_ and ΔSAR_1g_							
	Max SAR_1g_ [W/kg]	Mean SAR_1g_ [W/kg]	Max ΔSAR_1g_ [%]		Min ΔSAR_1g_ [%]		Mean ΔSAR_1g_ [%]
Without SR	5.77	1.27	Reference	Reference		Reference	
With SR (C_T-SR_ = 8.4 pF, opposing)	5.40	1.07	+ 219	− 101		− 10.2	
With SR (C_T-SR_ = 6.5 pF, enhancing)	7.25	1.47	+ 224	− 16.5		+ 9.81

**Table 2:** “Without SR” vs. “with SR (enhancing)” vs. “with SR (opposing)” (Ella at 128 MHz, 700 mm BP body coil, within VoI)

(A) Sternum					
	Max |B_1_^+^| [μT]	Mean |B_1_^+^| [μT]	Max Δ|B_1_^+^| [%]	Min Δ|B_1_^+^| [%]	Mean Δ|B_1_^+^| [%]
Without SR	5.60	2.96	Reference	Reference	Reference
With SR (C_T-SR_ = 12.0 pF, opposing)	5.54	2.76	+ 26.3	− 67.1	− 8.32
With SR (C_T-SR_ = 6.5 pF, enhancing)	7.96	3.37	+ 194	− 127	+ 17.7

	Max SAR_1g_ [W/kg]	Mean SAR_1g_ [W/kg]	Max ΔSAR_1g_ [%]	Min ΔSAR_1g_ [%]	Mean ΔSAR_1g_ [%]
Without SR	57.3	7.12	Reference	Reference	Reference
With SR (C_T-SR_ = 12.0 pF, opposing)	55.7	6.60	+ 152	− 42.3	− 4.76
With SR (C_T-SR_ = 6.5 pF, enhancing)	88.0	8.93	+ 717	− 36.1	+ 16.8

(B) Neck					
	Max |B_1_^+^| [μT]	Mean |B_1_^+^| [μT]	Max Δ|B_1_^+^| [%]	Min Δ|B_1_^+^| [%]	Mean Δ|B_1_^+^| [%]
Without SR	5.15	2.59	Reference	Reference	Reference
With SR (C_T-SR_ = 12.0 pF, opposing)	11.2	2.49	+ 2.7	− 128	− 7.52
With SR (C_T-SR_ = 6.5 pF, enhancing)	21.6	3.00	+ 428	− 156	+ 30.2

	Max SAR_1g_ [W/kg]	Mean SAR_1g_ [W/kg]	Max ΔSAR_1g_ [%]	Min ΔSAR_1g_ [%]	Mean ΔSAR_1g_ [%]
Without SR	71.7	7.23	Reference	Reference	Reference
With SR (C_T-SR_ = 12.0 pF, opposing)	67.5	6.80	+ 2422	− 376	− 17.1
With SR (C_T-SR_ = 6.5 pF, enhancing)	248	9.37	+ 9640	− 1752	+ 85.2

(C) Knee					
	Max |B_1_^+^| [μT]	Mean |B_1_^+^| [μT]	Max Δ|B_1_^+^| [%]	Min Δ|B_1_^+^| [%]	Mean Δ|B_1_^+^| [%]
Without SR	3.14	2.04	Reference	Reference	Reference
With SR (C_T-SR_ = 12.0 pF, opposing)	2.94	1.81	+ 81.5	− 116	− 20.8
With SR (C_T-SR_ = 6.5 pF, enhancing)	7.22	2.92	+ 457	− 119	+ 79.3

	Max SAR_1g_ [W/kg]	Mean SAR_1g_ [W/kg]	Max ΔSAR_1g_ [%]	Min ΔSAR_1g_ [%]	Mean ΔSAR_1g_ [%]
Without SR	12.9	2.26	Reference	Reference	Reference
With SR (C_T-SR_ = 12.0 pF, opposing)	26.5	2.06	+ 966	− 119	− 10.1
With SR (C_T-SR_ = 6.5 pF, enhancing)	39.8	3.31	+ 1698	− 52.4	+ 51.6

**Table 3:** |**B**_1_^+^|, Δ|**B**_1_^+^|, SAR_1g_, ΔSAR_1g_, SAR_10g_, and ΔSAR_10g_ “without SR” vs. “with SR (enhancing)” vs. “With SR (opposing)” (Ella with the copper rod at 128 MHz, 700 mm BP body coil)

	Mean |B_1_^+^| [μT]	Mean Δ|B_1_^+^| [%]	Mean SAR_1g_ [W/kg]	Mean ΔSAR_1g_ [%]	Mean SAR_10g_ [W/kg]	Mean ΔSAR_10g_ [%]
Without SR	3.26	Reference	22.9	Reference	23.6	Reference
With SR (C_T-SR_ = 12.0 pF, opposing)	2.87	− 12.9	19.3	− 18.3	21.7	− 20.4
With SR (C_T-SR_ = 6.5 pF, enhancing)	4.7	+ 45.1	27.9	+ 23.6	29.6	+ 52.6

**Table 4:** “Without SR” vs. “with SR (enhancing)” vs. “with SR (opposing)” (Duke at 128 MHz, 700 mm BP body coil)

(A) |B_1_^+^| and Δ|B_1_^+^|					
	Max |B_1_^+^| [μT]	Mean |B_1_^+^| [μT]	Max Δ|B_1_^+^| [%]	Min Δ|B_1_^+^| [%]	Mean Δ|B_1_^+^| [%]
Without SR	6.51	3.49	Reference	Reference	Reference
With SR (C_T-SR_ = 6.5 pF, enhancing)	6.48	3.28	+ 45.2	− 63.9	− 6.61
With SR (C_T-SR_ = 8.4 pF, opposing)	11.0	4.14	+ 186	− 85.3	+ 20.6

(B) SAR_1g_ and ΔSAR_1g_					
	Max SAR_1g_ [W/kg]	Mean SAR_1g_ [W/kg]	Max ΔSAR_1g_ [%]	Min ΔSAR_1g_ [%]	Mean ΔSAR_1g_ [%]
Without SR	93.1	10.4	Reference	Reference	Reference
With SR (C_T-SR_ = 6.5 pF, enhancing)	89.6	10.0	+ 113	− 76.2	− 6.19
With SR (C_T-SR_ = 8.4 pF, opposing)	201	13.6	+ 778	− 50.1	+ 15.1

All numerical simulations in this study were performed using the commercially available xFDTD software (Remcom, Inc.; State College, PA) and post-processing analysis was performed in Matlab (the MathWorks, Inc., Natick, MA).

### Experimental measurements

All experiments were performed using a 3 T MITS BP body coil (Zurich Med Tech, ID = 700 mm, inner length = 400 mm, outer length = 480 mm), an RF amplifier (ENI Inc., Richardson, TX, USA), an RF signal generator (Aeroflex Inc., Plainview, NY, USA), a fiber optic temperature measurement system (Neoptix Reflex), an ASTM phantom (length = 650 mm, width = 420 mm, and height = 90 mm. σ = 0.47 S/m and ε_r_ = 80), and designed SRs (Fig. 1b). The body coil was driven in a quadrature mode. The designed SRs had dimensions of ID ≈ 151 mm, OD = 160 mm, and a width of approximately 6 mm. They were fabricated using rectangular copper plates and tuned to frequencies of 125.02 MHz (C_T_SR_ = 6.5 + α pF, Opposing) and 135 MHz (C_T_SR_ = 5.0 + α pF, Enhancing) (Fig. 1b). The symbol α represents a capacitor value within a variable capacitor range of up to 15 pF. The EM field mapping probes enable the measurement of the RMS value of the EM fields’ amplitude but do not provide phase information. Consequently, it was not feasible to measure the values of |**B**_**1**_^**+**^|. All the experimental results were normalized to the same dissipated power.

## Results

Figure 1 shows geometrical models of a 16-leg BP birdcage transmit body coil with RF shield, the ASTM phantom, Ella, the SR, and the copper rod used in this study.

**Fig. 2:**
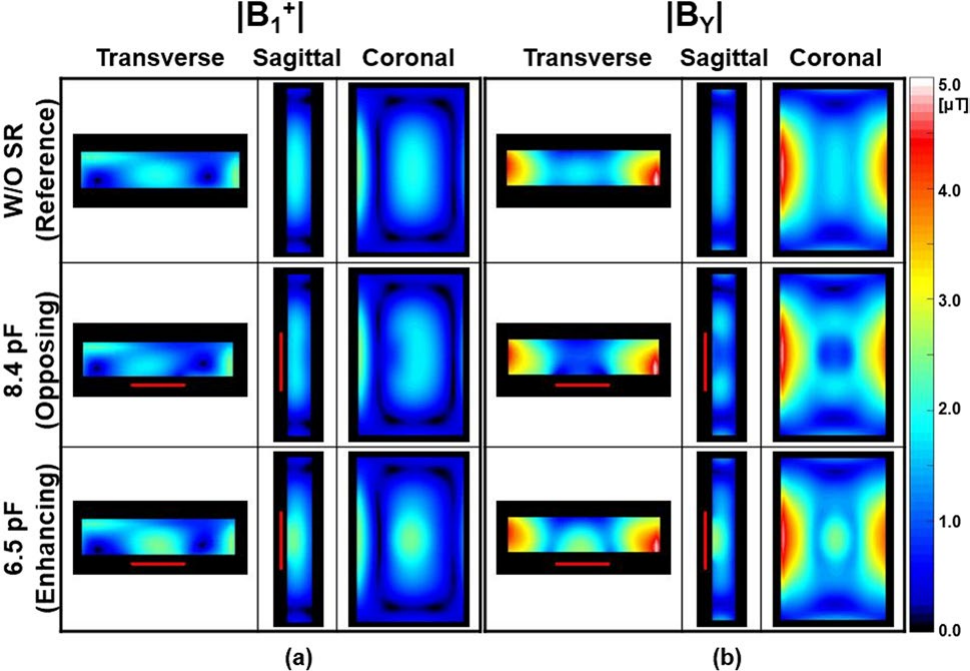
Numerical simulation results for |**B**_1_^+^| (**a**) and the magnitude of the magnetic flux density Y-component (|**B**_Y_|) (**b**) using the 16-leg BP birdcage coil within the ASTM phantom without (first row) and with the SR (second and third rows) at 3.0 T (Fig. 1a). The simulation results in this figure and Figs. 3 and 4 were normalized to the dissipated power of 48.84 W to make |**B**_1_^+^| equal to 2.0 µT at the center of the body coil without the SR. The red rectangular bars in the transverse and sagittal views indicate the location of the designed SR

**Fig. 3:**
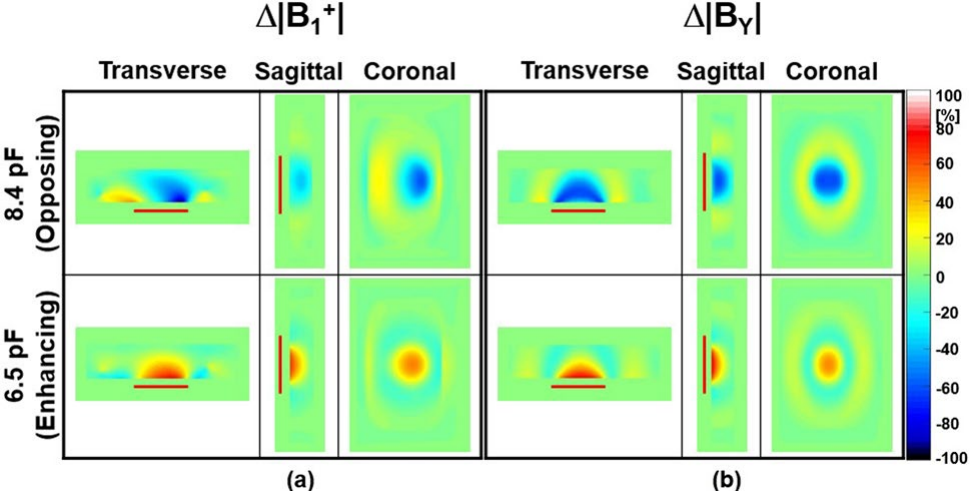
Numerical simulation results of the change of |**B**_1_^+^| (Δ|**B**_1_^+^|) (**a**) and |**B**_Y_| (Δ|**B**_Y_|) (**b**) within the ASTM phantom with the SR having a C_T-SR_ of 8.4 pF (opposing, first row) and 6.5 pF (enhancing, second row) at 3.0 T. Other parameters are same as in Fig. 2

**Fig. 4:**
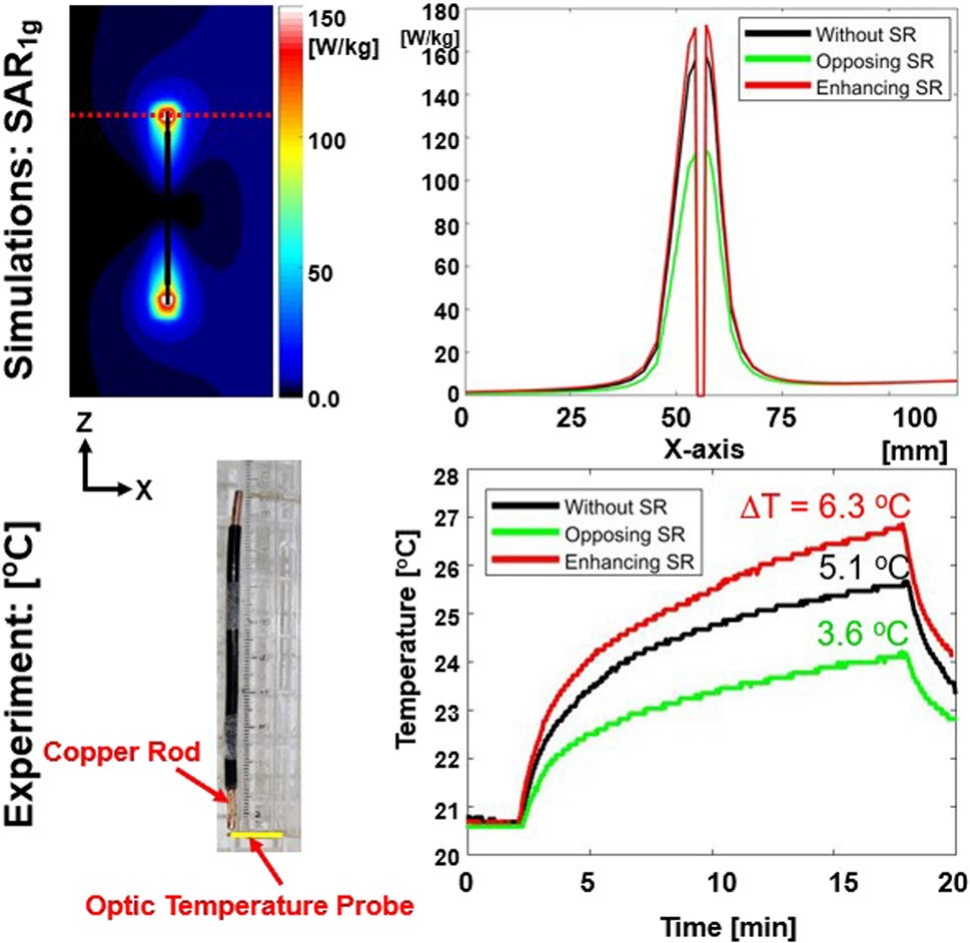
Numerical simulations (first row) of SAR1g and corresponding experimental results of temperature measurement (second row) near the tip of a copper rod using optic fibers without (black line) and with designed SRs of opposing (green line) and enhancing (red line). The red dotted rectangular lines in the first row indicate the location of the 1D profile

Figures 2, 3, and 4 show numerical simulation results of |**B**_1_^+^|, |**B**_Y_| (Fig. 2), Δ|**B**_1_^+^|, Δ|**B**_Y_| (Fig. 3), and **SAR**_**1g**_ (Fig. 4, first row) without and with the SRs using the ASTM phantom and 700 mm BP body coil. The corresponding experimental results of temperature rise using the designed SRs and ASTM phantom were shown in Fig. 4 (second row). The detailed information of max, min, and mean values of |**B**_1_^+^| and **SAR**_**1g**_ are shown in Table 1. The SR with C_T-SR_ of 8.4 pF makes opposing |**B**_Y_| and |**B**_1_^+^| (second row in Fig. 2), whereas the SR with C_T-SR_ of 6.5 pF makes enhancing magnetic fields of |**B**_Y_| and |**B**_1_^+^| (third row in Fig. 2) compared to the magnetic fields made by the body coil (without the SR, first row in Fig. 2). The effect of opposing or enhancing magnetic fields made by the SR is more obvious in Δ|**B**_Y_| than Δ|**B**_1_^+^| (Fig. 3). The reason is because the **B**_1_^+^ is combination of **B**_**X**_ and **B**_Y_ [28], and **B**_**X**_ is not designed to produce the desired magnetic fields in this specific design of the SR. The performance can be enhanced if the design of the SR is modified to a more optimized form, such as phased array for the target fields. However, under these simulation conditions, the SR primarily functions, albeit at the cost of some unwanted interactions meaning it can yield opposite results in certain regions, such as increased Δ|**B**_1_^+^| and Δ|**B**_Y_| when using an opposing SR, as shown in Fig. 3 and Table 1.

The temperature rise results in Fig. 4 (second row) with three different conditions of without (5.1 °C), with an opposing SR (3.6 °C), and with an enhancing SR (6.3 °C) are well matched with the results of **SAR**_**1g**_ numerical simulations (Fig. 4 first row). It is consistent with previous research [29] showing the temperature change using the ASTM phantom is proportional to the **SAR** (ΔT ∝ **SAR**).

**Fig. 5:**
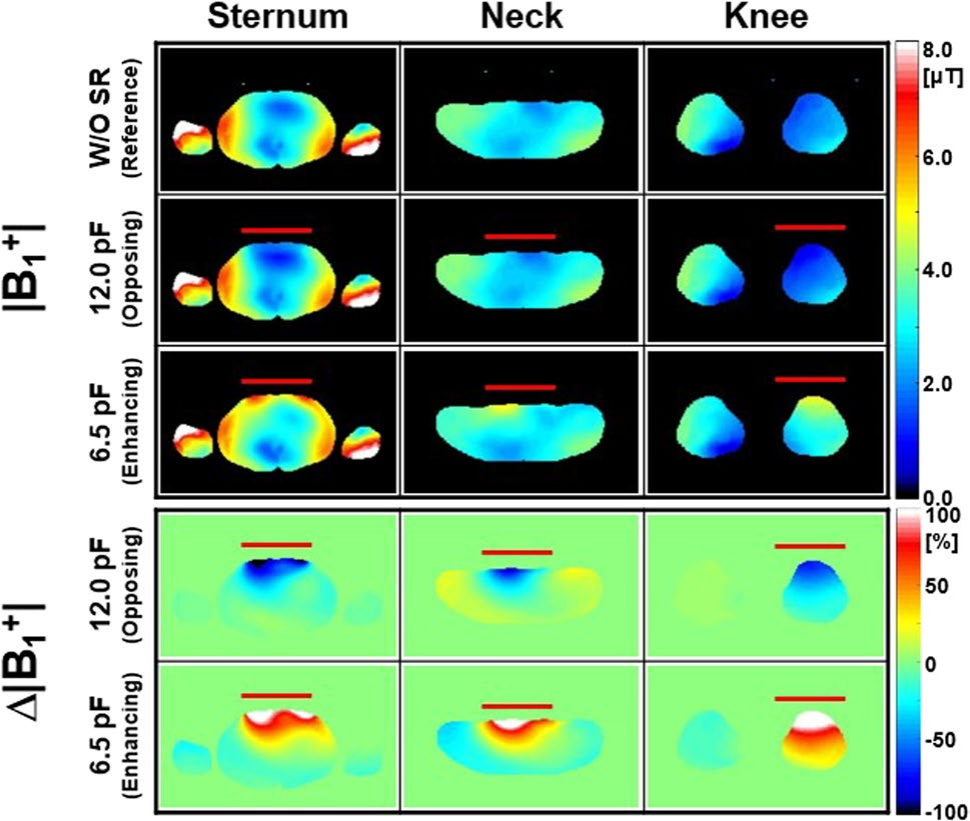
Numerical simulation results for |**B**_1_^+^| and Δ|**B**_1_^+^| within the Ella model under various conditions: without SR (first row), with opposing SR (second and fourth rows), and with enhancing SR (third and fifth rows) at different landmark positions—sternum (first column), neck (second column), and knee (third column) at 128 MHz (Fig. 1d–f). The red rectangular bars indicate the locations of the designed SR

**Fig. 6:**
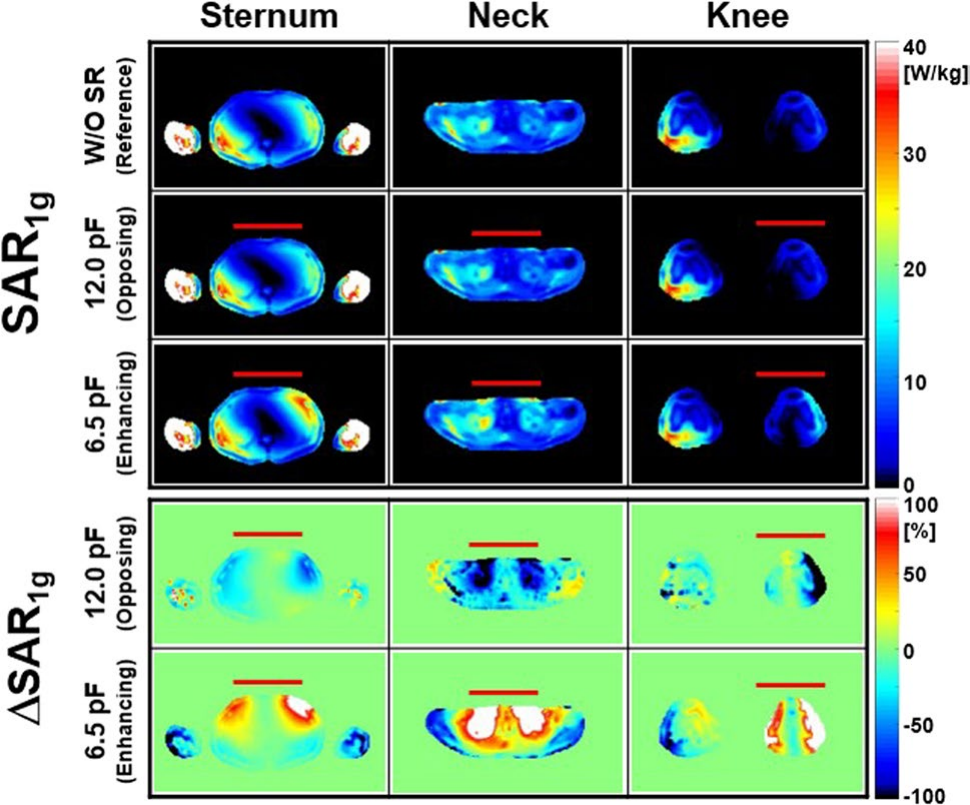
Numerical simulation results of **SAR**_**1g**_ and Δ**SAR**_**1g**_ within the Ella model with different landmark positions. Other parameters are same as in Fig. 5

Figures 5 and 6 show numerical simulation results of |**B**_1_^+^|, Δ|**B**_1_^+^| (Fig. 5), **SAR**_**1g**_ and Δ**SAR**_**1g**_ (Fig. 6) within the Ella model at different landmark positions of Sternum (first column), Neck (second column) and Knee (third column) at 128 MHz (Table 2). The C_T-SR_ for making enhancing magnetic fields was 6.5 pF which is same as that in the ASTM phantom. However, it was changed to 12.0 pF to create opposing magnetic fields.

A single plane of transverse data was displayed to save space (Figs. 5 and 6). The effect of the SR was more prominent at the Neck and Knee landmarks than at the Sternum landmark. For example, the mean **ΔSAR**_**1g**_ values were − 17.1% (Neck landmark) and − 10.1% (Knee landmark), while it was − 4.76% (Sternum landmark) when the SR created opposing magnetic fields (Table 2). This is probably because the region of Neck and Knee landmarks is smaller than that of Sternum landmark making more portion of the region with the effect of the SR and less interaction with unwanted EM-fields made outside region of the SR (Figs. 5 and 6, Table 2).

**Fig. 7:**
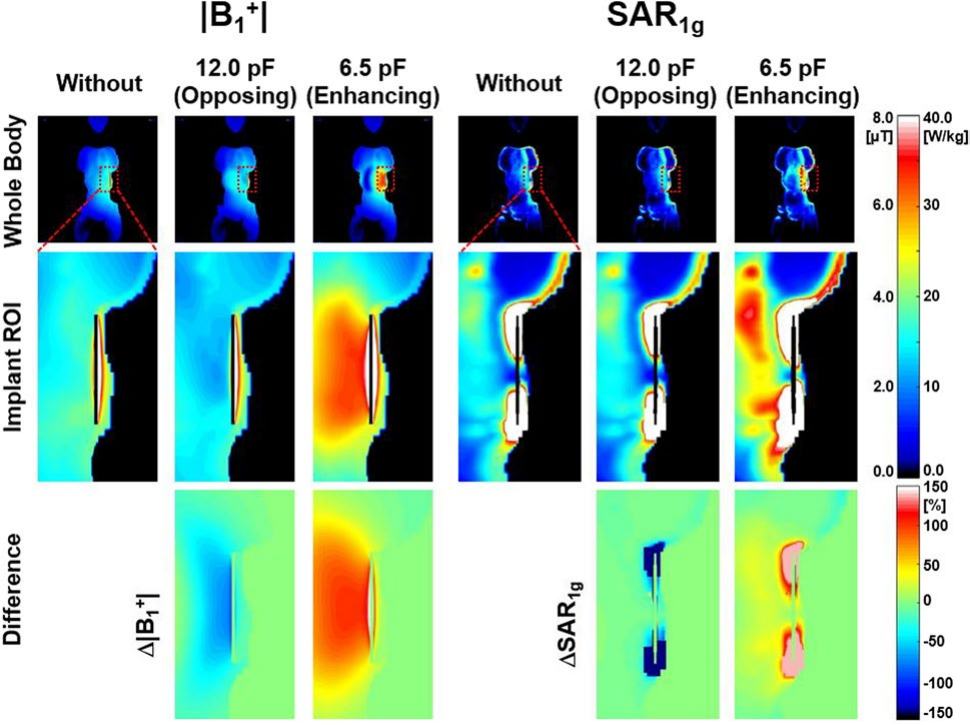
Numerical simulation results of |**B**_1_^+^| (left three columns) and **SAR**_**1g**_ (right three columns) within the Ella model near the copper rod representing broad spectrum of medical implants with opposing (C_T-SR_ = 12.0 pF, second and fifth columns) and enhancing (C_T-SR_ = 6.5 pF, third and sixth columns) SRs at 3.0 T. Center coronal views of copper rod within a whole body (first row), implant *VoI* (second row), and difference within the implant *VoI* (Δ|**B**_1_^+^| and Δ**SAR**_**1g**_, third row) (Fig. 1g). Multiple grid resolutions of 0.5 × 0.5 × 0.5 mm^3^ (copper rod region) and 3 × 3 × 3 mm^3^ (others) were used for the numerical simulations

Figure 7 shows numerical simulation results of |**B**_1_^+^| (within the whole body and implant *VoI*), Δ|**B**_1_^+^|, **SAR**_**1g**_ (within the whole body and implant *VoI*) and Δ**SAR**_**1g**_ within the Ella model near the copper rod representing medical stents to evaluate the effect of designed SR. with the SR making opposing magnetic fields within the *VoI* (132 × 132 × 135 mm^3^), which is not so big (Table 3).

**Fig. 8:**
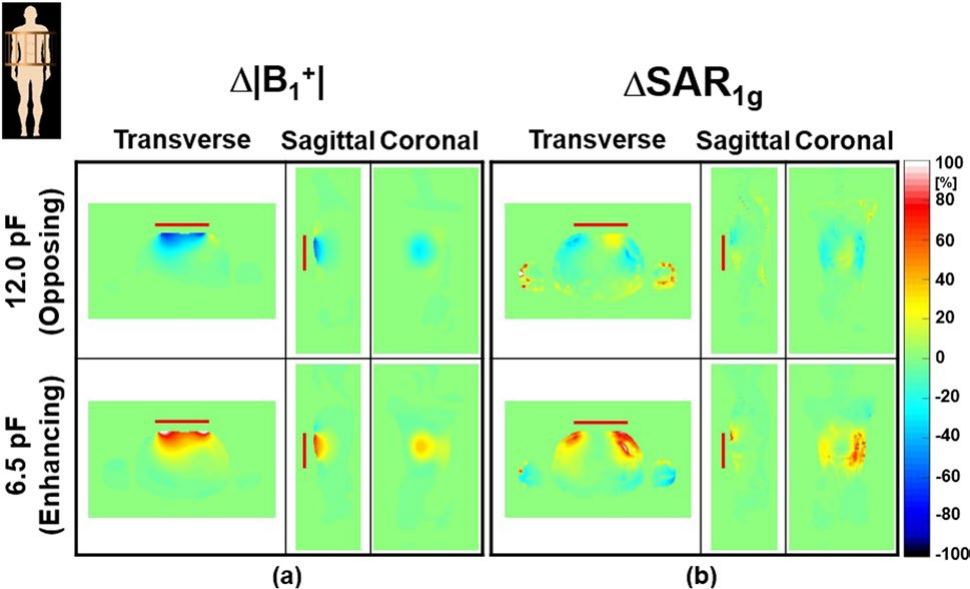
Numerical simulation results of Δ|**B**_1_^+^| (**a**) and Δ**SAR**_**1g**_ (**b**) within the Duke model with opposing (first row) and enhancing (second row) SRs at 3.0 T. The simulation results in this figure were normalized to the dissipated power of 445.1 W to make |**B**_**1**_^**+**^| equal to 2.0 µT at the center of the body coil without the SR

Figure 8 and Table 4 show the effectiveness of the designed SR using the Duke model at 3.0 T. Compared to the Ella model results, the same C_T-SR_ of 6.5 pF (Enhancing) and 12.0 pF (Opposing) were used for the numerical simulations. The Min Δ**SAR**_**1g**_ was − 76.2% with the SR making opposing magnetic fields within the *VoI* (Table 4), which is smaller than that of the Ella model (− 42.3% in the Sternum landmark).

## Discussion

The primary innovation of this study lies in proposing a new method that uses a SR designed to create opposing magnetic fields and lower SAR distributions. To substantiate this method, numerical simulations of different conditions at 128 MHz, along with corresponding experimental verifications using the designed SRs and the ASTM phantom, were conducted.

The theoretical basis of the proposed method is that the magnitude of the magnetically induced electric field (${{\varvec{E}}}_{{\varvec{i}}}$) is proportional to the time derivative of **B**_**1**_, as outlined in Eq. (3). Thus, if the SR can reduce **B**_**1**_ by creating opposing magnetic fields, it would decrease ${{\varvec{E}}}_{{\varvec{i}}}$, resulting in a reduced **SAR** (since **SAR** ∝|**E**|^2^) and improved RF safety within the *VoI*, assuming the conservative electric field, another component of the total electric field (${{\varvec{E}}}_{{\varvec{T}}}$), does not significantly change [30–32]. However, separating ${{\varvec{E}}}_{{\varvec{i}}}$ from ${{\varvec{E}}}_{{\varvec{T}}}$ at high frequencies can be challenging due to the wavelength effect [30]. As such, this study used the **SAR** data of **SAR**_**1g**_ and **SAR**_**10g**_, rather than ${{\varvec{E}}}_{{\varvec{i}}}$, to demonstrate the SR's effect on improving RF safety within the *VoI*.

Another way to explain our proposed method is that the equivalent circuits of the SR can be represented as either a capacitor or an inductor, each exhibiting a 180° phase difference in impedance. This means that the SR can be adjusted to either enhance **B**_**1**_ by creating in-phase magnetic fields in comparison to those generated by the body coil, or to oppose **B**_**1**_ by producing magnetic fields in the opposite direction, depending on the tuning status of the SR.

The effectiveness of the SR is highly dependent on the uniformity of the electromagnetic field created by the body transmit coil and the designed SR in the region of interest (ROI). Theoretically, the electromagnetic fields generated by the SR, when set to opposing fields in the ROI, should have the same magnitude as, but a 180° phase shift from, the fields created by the body transmit coil. Therefore, if the body coil's field uniformity in the ROI is suboptimal, designing an appropriate SR would be challenging. However, in situations where there is a relatively uniform and one-directional electromagnetic field component, such as fields created by surface coils, designing a suitable SR would be comparatively easier. Specifically, the body coil used in this study operated in quadrature mode, generating RF magnetic fields of **B**_**X**_ and **B**_**Y**_, as well as a Z-component of the RF magnetic field (**B**_**Z**_), which was primarily produced by the end rings of the body coil and considered an unwanted component in this study. In contrast, the designed RF magnetic fields created by the SR were predominantly **B**_**Y**_ in this study.

As a result, interactions between RF magnetic fields generated by the SR and those produced by the body coil (**B**_**X**_ and **B**_**Z**_) could lead to undesirable EM-field distributions. This might be the main reason that the effect of the SR was less pronounced in some regions (Figs. 2, 3, 4, 5, 6, 7 and 8). The effect of the SR was more noticeable at |**B**_Y_| than at |**B**_1_^+^| for the same reason (Figs. 2 and 3). This issue can be addressed by modifying the design of the body coil or the SR in future studies.

The landmark positions of the neck, sternum, and knee were chosen with specific implants in mind, such as cervical implants for the neck region, pacemakers and breast tissue expander devices [6] for the sternum area, and knee replacement implants for the knee.

Some parameters used in the study, such as the ID and tuning capacitor values of the designed SRs, as well as the distance between the SRs and the ASTM phantom or human models, were selected following a coarse parametric optimization (results not included here). Further optimization tailored to specific applications will be performed in future studies.

Our proposed method can be used to attenuate the **SAR** or signal using a designed SR tailored for specific regions. One example could be to target the high **SAR** region of a medical stent implant, which would not be located at the main imaging region of the MRI system. Another application could be to attenuate signal from regions that can fold over into the active Field of View (FOV), such as for the signal from the contra lateral knee in small FOV knee MRI (Figs. 5 and 6). In routine use, one could envision adding additional surface coils designed for signal attenuation in addition to the coils for signal reception.

The limitations of our proposed method could include (1) the difficulty in designing an SR when the |**B**_**1**_^**+**^| distribution within the *VoI* is not uniform, and (2) potential MR image artifacts caused by the EM-fields of the SR when the *VoI* is close to the MR imaging region.

## Conclusions

In conclusion, we have proposed a novel method using a specifically designed SR that generates opposing magnetic fields to partially shield a sample, thereby improving RF safety at the *VoI*. Data supporting this hypothesis was obtained through numerical simulations under different conditions at 3.0 T, and this was further substantiated through experimental verification. Our proposed method and findings can provide valuable insights for the evaluation and improvement of RF safety in high-field MRI.

## Data Availability

The datasets generated and analyzed during the current study are available from the corresponding author on reasonable request.

## Declarations

### Disclaimer

The mention of commercial products, their sources, or their use in connection with material reported herein is not to be construed as either an actual or implied endorsement of such products by theUS Food and Drug Administration.
